# The importance of personal documentation for patients living with long‐term illness symptoms after pituitary surgery: A Constructivist Grounded Theory study

**DOI:** 10.1111/hex.13648

**Published:** 2022-11-06

**Authors:** Birgit Heckemann, Tatjana Graf, Eva Jakobsson Ung, Sofie Jakobsson, Oskar Ragnarsson, Daniel S. Olsson, Christina Blomdahl

**Affiliations:** ^1^ Section for Care in Long‐term Conditions, Institute of Health and Care Sciences, Sahlgrenska Academy University of Gothenburg Gothenburg Sweden; ^2^ Department of Anesthetics, Surgery and Intensive Care Sahlgrenska University Hospital Gothenburg Sweden; ^3^ Department of Sociology University of Lucerne Lucerne Switzerland; ^4^ Section for Learning and Leadership for Health Care Professionals, Institute of Health and Care Sciences, Sahlgrenska Academy University of Gothenburg Gothenburg Sweden; ^5^ Department of Medicine Sahlgrenska University Hospital Gothenburg Sweden; ^6^ Department of Internal Medicine and Clinical Nutrition, Sahlgrenska Academy, Institute of Medicine University of Gothenburg Gothenburg Sweden; ^7^ Research and Development Centre Södra Älvsborg, Research and Development Primary Healthcare Region Västra Götaland Borås Sweden

**Keywords:** chronic disease, chronic illness, Constructivist Grounded Theory, patient documentation, personal documentation, person‐centred care, pituitary adenoma

## Abstract

**Introduction:**

Despite surgical treatment, pituitary adenomas often cause long‐term illness symptoms, that profoundly impact patients' quality of life physically, psychologically and socially. Healthcare professionals often fail to recognize and discuss the ensuing problems. Personal documentation, such as symptom monitoring, reflective writing or even posts on social media, may help this patient group to manage their daily life and support communication of their care needs. Documentation strategies and the role of documentation for people with long‐term symptoms after pituitary adenoma surgery are currently unknown.

**Aim:**

To examine the effects and strategies of documenting symptoms, activities and physical and emotional well‐being among people living with long‐term pituitary adenoma.

**Methods:**

In this Constructivist Grounded Theory study, 12 individuals living with long‐term illness symptoms after pituitary adenoma surgery described their documentation strategies in in‐depth interviews using teleconferencing and photo‐elicitation between August and October 2020.

**Results:**

Strategies for documentation included analogue and digital media. One core category (Exercising autonomy) and three categories describing processes (Gaining insight, Striving for control and Sharing) emerged from the analysis. These three interrelated processes become an expression of autonomy to manage life and make sense of chronic illness. Personal documentation is a flexible tool that is used more extensively in times of ill health and less in times of relative well‐being. Sharing documentation with healthcare professionals facilitated care planning and sharing with friends and family fostered emotional well‐being.

**Conclusion:**

Personal documentation is a valuable resource for managing life after pituitary adenoma surgery. The current findings may be relevant to other chronic illnesses. Further research exploring potential tools for personal documentation is needed.

**Patient or Public Contribution:**

We deliberately chose a Constructivist Grounded Theory approach for this interview study. Using Constructivist Grounded Theory, we gave people living with long‐term symptoms a voice, allowing them to freely speak about managing their illness in connection with personal documentation. The theoretical sampling approach enabled us to invite participants that could provide a broad overview of the landscape of personal documentation.

## 1 INTRODUCTION

Pituitary adenomas are rare^1^, ^2^ but can entail a heavy symptom burden and a chronic illness course despite surgical treatment.^3^, ^4^ Long‐term illness symptoms can vary significantly between patients depending on the type of adenoma.^3^ Patients with acromegaly typically develop progressive changes in facial appearance and growth of the hands, feet and internal organs.^5^ Cushing's syndrome often causes proximal muscle weakness, fatigue and sleeping problems, central obesity, a round face and dorsocervical fat accumulation. Comorbidities with Cushing's syndrome include hypertension, diabetes, infections and hypogonadism.^6^ Prolactinoma typically causes infertility.^7^ Long‐term health‐related overall quality of life is often reduced in patients with pituitary adenoma.^8^ Pituitary adenomas are typically treated with surgery, medical therapy and potentially radiotherapy, except for prolactinomas, which are usually treated with medication.^9^

There is a robust body of quantitative research exploring the quality of life of this patient group.^3^, ^5^ While patients' health‐related quality of life may increase initially after surgery, it often fails to normalize in the long term.^8^ Depending on the underlying condition, long‐term symptoms can include joint pain or headaches, hypertension, memory deficiencies or fatigue.^8^ Qualitative studies have reported that patients often live with unpredictable symptoms, cognitive problems such as attention, concentration and memory problems, altered physique, sexual dysfunction, fatigue, pain and psychological complaints such as depressive symptoms, anxiety, stress and fear.^10^, ^11^ Personality‐related issues may occur, including personality changes and altered emotional functioning. Patients report work and social problems, including changes in social functioning, adverse effects of the illness on personal relationships, reduced social networks and a general lack of sympathy and support.^4^, ^10^

Despite the heavy symptom burden associated with the condition, qualitative research examining the experiences of living with long‐term illness symptoms after pituitary adenoma surgery is scarce. Several recent studies have highlighted that patients' care needs are often not adequately met. Healthcare professionals may lack specialist knowledge or fail to recognize and discuss issues regarding sexuality, fatigue and other psychological or personal problems that impinge on patients' lives^4^, ^10^, ^11^ Patients require structured, continued support that addresses their physical, cognitive and existential challenges.^4^

Personal documentation may play an important role in improving care and self‐care in this patient group. The benefits of various types of personal documentation in managing chronic illness are well known. For example, monitoring improves the control and day‐to‐day management of physical symptoms, such as blood sugar levels or blood pressure.^12^, ^13^, ^14^ Recently, there has been increased interest in Patient‐Generated Health Data (PGHD) to improve chronic illness treatment. PGHD are any health‐related data generated by a patient, such as ‘biometric data, symptoms, lifestyle choices, and treatment history’.^15^ With PGHD, patients are responsible for collecting and choosing with whom they share their data.^15^ However, although the approach is patient‐oriented, this documentation focuses on biomedical information. Other forms of documentation, such as expressive writing, can be more therapeutic, as they support physical and psychological health.^16^ Narration and storytelling facilitate making sense of traumatic or stressful experiences.^17^, ^18^ Emotional catharsis, narration and storytelling can reduce psychological stress and contribute to developing a coherent life narrative.^16^

For the current study, we drew on Plummer's^19^ seminal work to define personal documentation from a sociological angle. Plummer^19^,p.17 writes: ‘People keep diaries, send letters, make quilts, take photos, dash off memos, compose auto/biographies, construct websites, scrawl graffiti, publish their memoirs, write letters, compose CVs, leave suicide notes, film video diaries, inscribe memorials on tombstones, shoot films, paint pictures, make tapes and try to record their personal dreams. All of these expressions of personal life are […] in the broadest sense' documents of life’. Based on these deliberations, we defined personal documentation to include narrative writing and journaling but also the recording of symptoms or activities or creating images. In line with this definition, we regarded documentation not in a generic sense but as an expression of different processes, for example, individual reflection and social network engagement.

Although previous evidence suggests that various documentation techniques may be valuable resources, the roles of documentation and the documentation strategies employed by patients in managing a condition with diverse chronic symptoms, such as pituitary adenoma, are largely unknown. This study explored patients' strategies for documenting information about symptoms and physical and emotional health in a life affected by chronic symptoms following pituitary adenoma surgery.

## 2 METHOD

### 2.1 Design

This qualitative interview study used a Constructivist Grounded Theory approach.^20^ We chose this approach because it enabled us to understand the meaning of personal documentation in people living with a pituitary adenoma. In contrast to other qualitative research approaches, the Constructivist Grounded Theory approach permitted us to study personal documentation in great depth, explore its role concerning individual and social life and generate a theory about this scantly investigated phenomenon.^21^

### 2.2 Study context

This interview study was conducted in the context of a larger quasi‐experimental study designed to evaluate the implementation of a person‐centred care pathway for patients receiving treatment for pituitary adenoma.^22^ The interview study population comprised 47 patients who participated in the trial's intervention group between December 2017 and May 2019.

### 2.3 Sample and sampling

**Figure 1:**
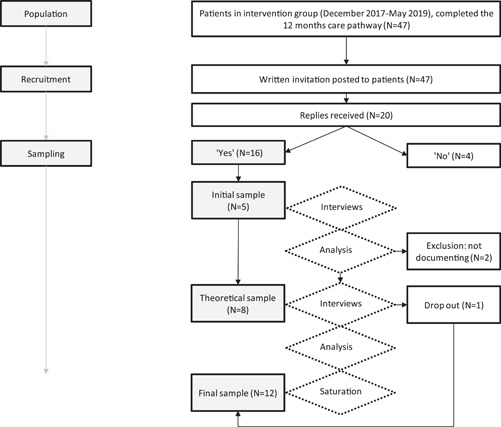
Recruitment and sampling process

We invited 47 patients that had undergone pituitary adenoma surgery to participate in the study. Twenty of the 47 patients replied, and 16 were interested in participating in the study. In line with the Constructivist Grounded Theory approach,^20^ we included five participants during the initial sampling phase. These were the patients that had replied first. We selected eight additional participants during the theoretical sampling phase and excluded two further participants because they did not perform documentation. One participant dropped out of the study, resulting in a final sample of 12 participants (six women; six men) (Figure 1). The age range was 35–58 years (mean 54 years). Table 1 shows the characteristics and demographics of the study participants.

**Table 1:** Participant characteristics and demographics

Characteristics and demographics (*N* = 12)	Number of participants
Gender	
Female	6
Male	6
Age	
30–50	5
51–80	7
Diagnosis	
Nonfunctioning pituitary adenoma	5
Acromegaly	4
Cushing	1
Prolactinome	1
Rathke's cyst	1
Operation	
First	7
Reoperation	5
Country of birth	
Sweden	11
Other European country	1
Educational level	
High school/vocational training	6
Middle school	2
University	4
Occupation	
Working	8
Other	4
Living situation	
Cohabiting	9
Living alone	3

Except for the first two interviewees, all participants either kept personal documentation and/or read their official personal online health records, which are accessible to patients in Sweden. Keeping personal documentation or reading their online health record became one of our theoretical sampling criteria because it became apparent during the analysis process that personal documentation played a complex and multifaceted role in managing life with a chronic illness.

### 2.4 Data collection

The interviews took place between August and October 2020, using teleconferencing and photo‐elicitation,^23^ which enabled safe data collection during the coronavirus‐2019 pandemic. One co‐author (C. B.), with substantial experience in arts‐inspired face‐to‐face interviewing, conducted the interviews. All interviews were conducted in Swedish. We used ‘Mitt vårdmöte’ (My Care Meeting), an application that enables safe healthcare online consultations, to protect our participants' anonymity. The app is a CE‐marked (CE marking indicates conformity with health, safety, and environmental protection standards for medical technology products sold within the European Economic Area. https://eurlex.europa.eu/LexUriServ/LexUriServ.do?uri=CELEX:31993L0068:en:HTML).  The app complies with the Patient Data Act, the General Data Protection Regulations (GDPR) and the Swedish Data Protection Authority's rules.^24^ The videoconferencing technology enabled participants to freely choose a place from which to join C. B. for the interview. A set of introductory questions such as ‘Do you document in daily life?’, ‘Can you tell me about how you document?’ or ‘Which information about your symptoms or health in your daily life is important?’ were used in the opening phase of the interviews. Further questions were generated from the concurrent analysis and the participants' narratives. In line with our definition,^19^ we aimed to encourage the participants to reflect broadly on their documentation activities. We invited participants to look at 20 photos of objects related to writing (e.g., a typewriter, a computer, a bookshelf, a mobile phone and headphones) and scenes that conveyed different moods that could be linked to the illness experience. All images were from B. H.'s private stock. Photo‐elicitation is an established method for stimulating interview participants' thinking, encouraging storytelling, fostering reflection and facilitating free association.^25^, ^26^, ^27^, ^28^ Photo‐elicitation is particularly suited to exploring activities, interactions and processes.^26^

The interviews lasted for 18–78 min (mean 49 min), and were digitally recorded and transcribed verbatim.

### 2.5 Data analysis

According to the Constructivist Grounded Theory approach, the interview transcripts were analysed concurrently during the data collection phase.^20^ We used a constant comparative method to analyse the data; B. H. and T. G. were the main coders. B. H. and T. G. coded independently, but they had 17 weekly meetings, each lasting 60–90 min during the analysis, to discuss and compare the codes and their meanings.

The coding process for each interview included initial reading and rereading of the transcript and open coding of the texts. We did not code line‐by‐line but instead moved straight to coding meaning units, which provided deep insights. We labelled text excerpts with codes or phrases that were as close to the original meaning as possible, thus maintaining a low level of abstraction. During the coding process, we constantly compared meanings and identified similarities and differences within and between the interviews, continuously moving to more abstract and more specific codes, categories and concepts.^23^, ^29^ We also recorded reflections, questions and ideas in memos. B. H. and T. G. used the memos to inspire the exploration of  additional literature to broaden their horizons and maintain a questioning mind to better understand the possible meaning of the data. The authors B. H., S. J., E. J. U., C. B. and T. G. met four times to discuss the analysis. This collaborative approach enabled critical discussion, spawned ideas, identified gaps in data collection and clarified the relationships between the codes and categories. As a team, we decided that we had reached saturation after 12 interviews.^20^ We used Nvivo software,^29^ Word documents and Excel spreadsheets to manage, analyse and visualize the data.

### 2.6 Quality criteria

Credibility, originality, resonance and usefulness have been proposed as quality criteria for Constructivist Grounded Theory research.^20^, ^30^

We ensured credibility with a theoretical sampling process guided by concurrent data analysis. After 12 interviews, we closed the data collection because we had reached a saturation of categories (instead of data),^20^ not due to lack of participants or resources. Our study is original in that it explores documentation from a purely patient‐driven angle. This angle extends the current ideas about patient documentation, which is often symptom oriented instead of capturing the lived experience. Resonance pertains to how the study relates to larger collectives and individual lives. The study's usefulness relates to its contribution to knowledge, further research and generic processes. We discuss the resonance and usefulness in the discussion section of this paper (transferability).

### 2.7 Reflexivity statement

A reflexive stance is essential in Constructivist Grounded Theory.^20^, ^30^ In the following paragraphs, we briefly outline our reflections drawing on Gentles et al.^31^

#### 2.7.1 Researchers' influence on research design and decisions

B. H., E. J. U. and S. J. initiated this project because of their professional and personal interest in documentation, person‐centred care and chronic illness. Sharing a nursing background, we felt compelled to highlight this often‐overlooked patient group's experiences and challenges in the research community and clinical practice. The initial decisions regarding the research topic, research questions and methodology were taken jointly during meetings and ongoing discussions. The remaining authors joined at the later stages. All authors contributed to the data analysis, interpretation and manuscript writing using their different professional lenses from occupational therapy (C. B.), sociology (T. G.) and medicine (D. S. O. and O. R.). We considered this input from different angles to be important because we did not want to produce research that was biased towards a nursing agenda.

#### 2.7.2 Researcher–participant interactional influences during data collection

C. B. collected the interview data and was in email contact with the participants before the interviews. Having a background in occupational therapy and mental health and no prior knowledge about this patient group, C. B. interviewed participants from a place of curiosity, using empathy to establish rapport. C. B. evolved as a researcher during the data collection. The first few interviews taught her about the experience of life after pituitary adenoma surgery. During the later interviews, she immediately related to participants' experiences, which enabled deeper discussion and co‐construction of meaning. This evolution affected the power balance during the interviews. Because C. B. was initially inexperienced regarding the condition, the power balance tipped towards the participants, who held specialist knowledge. With her increasing experience, the power balance shifted towards equality, which opened space for co‐constructing meaning and interpretation in the interviews.

#### 2.7.3 Researchers' influence on the analysis

The data analysis was a team effort. We discussed and interpreted the data through the lenses of nursing, occupational therapy, mental health and sociology. We ensured theoretical sensitivity through several measures: C. B. and B. H. discussed their thoughts about each interview. B. H. and T. G. kept memos on their thoughts, feelings and insights. B. H. and T. G. frequently met to discuss coding and data visualizations. While we explored participants' experiences in‐depth, we also continuously reflected on the meaning and application of the data within the broader context of health care and society.

#### 2.7.4 Researchers' influence on the writing

We designed this study to improve visibility and care for this patient group. We conducted this study from a place of empathy, which might have biased our writing. However, we feel that this stance was also essential to do justice to the participants' experiences.

#### 2.7.5 Influence of the research on the researcher

This research gave the research team a heightened understanding of living with long‐term symptoms after pituitary adenoma surgery and patients' resourcefulness in managing their challenges with the aid of documentation.

### 2.8 Ethical considerations

Approval of the study was obtained from the Swedish Ethical Review Board (DNR 2019‐06485 and 2020 03025). The data collection complied with the Declaration of Helsinki principles.^32^ All data were de‐identified. Data were stored on a secure university server and only accessible to members of the research team. Participants received written and verbal information about the study and provided written informed consent before the interviews.

## 3 RESULTS

### 3.1 Strategies for documentation

Many participants in our study used analogue media, including health diaries (as part of a care plan), diaries, calendars, notebooks or scraps of paper to document. Others used digital media, such as mobile phones or digital applications. Some posted on social media such as Instagram or Facebook, and some used a combination of analogue and digital media. Younger participants (35–40 years old), were more inclined to use digital media compared with older participants. The documentation content oscillated between mundane scribbles and quick, spontaneous note‐taking about symptoms or activities in calendars, to more elaborate, reflective writing to preserve memories or release emotions. Some participants used images such as emojis to express feelings. Others took photographs to document their well‐being or physical changes over time. Participants who struggled with the emotional burden of the illness tended to write more reflectively and elaborately.

**Figure 2:**
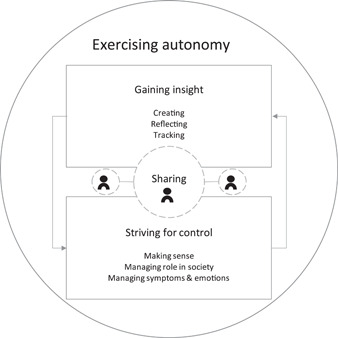
Exercising autonomy through personal documentation

In the following paragraph, we describe the theory that emerged from our analysis. The theory explains how three interrelated processes (described in categories) enable *Exercising autonomy* in managing life with a chronic illness (Figure 2).

### 3.2 Core category: Exercising autonomy

Healthcare services are essential for the adequate medical management of chronic illnesses. However, services such as clinic appointments are often scheduled at spread‐out intervals of weeks or months, leaving patients to manage the effects of a chronic illness on their daily lives on their own in the interim. Our analysis showed that personal documentation plays an important role in the autonomous management of chronic illness, as it has diverse functions. The written texts or images mirror thoughts, feelings, experiences and situations, thus enabling new understanding or insights and an opportunity for self‐reflection.

Oddly enough, I think that's what feeling bad is all about: That is why I felt bad and that's what made me feel bad. And things like that, and what happened, or how I experienced it. I think that's quite interesting, because you learn from your mistakes […]. (PT 8)

Self‐reflection is an opportunity to ponder and understand oneself and one's situation in a novel way. Enhanced knowledge of the self provides access to strategies to deal with the illness, make health decisions or cope with life's challenges. The results revealed that patients use documentation creatively. Additionally, patients were mostly self‐motivated but sometimes inspired by others, such as family or healthcare professionals.

[…] I got a great little book from my daughter. She had written nice things in it, some of her own things, a little here and there. And I'd read it a number of times, but never added anything. But about two weeks ago […], I started writing in the book […]. (PT6)

The core category comprised three interconnected categories describing processes in personal documentation. One category concerns *Striving for control* in one's social role, the second relates to *Gaining insight* about life with chronic symptoms, and the third regards *Sharing* personal documentation. The activities associated with *Gaining insight* provide the basic information to enable *Striving for control*. The third category, *Sharing*, lies at the intersection of *Gaining insight* and *Striving for control*. *Sharing* documentation with healthcare professionals can support care planning; sharing documentation with loved ones can foster feelings of trust, belonging and connection. However, our interview analysis did not enable us to determine whether these processes were sequential or of equal importance.

### 3.3 Categories

*Gaining insight* is a process that comprises actions including (1) Creating, (2) Reflecting and (3) Tracking. These actions capture current information about different aspects of life, such as symptoms, emotional states or events.

In *Striving for control*, these pieces of information are aggregated to enable (1) Making sense, (2) Managing Symptoms and Emotions, as well as (3) Managing (one's) role in society. The third category, *Sharing*, lies at the intersection of the two categories. *Sharing* selected pieces of documentation with specific, trusted persons (family, friends, healthcare professionals) can increase the benefit of personal documentation: *Sharing* with loved ones can foster feelings of proximity, whereas sharing symptom diaries with healthcare professionals can enable diagnosis or care and treatment planning.

#### 3.3.1 Gaining insight

Three actions supported the process of *Gaining insight*.

(1) Creating
  Expressing oneself through creating images or text can preserve personal memories or a legacy, or simply give a sense of achievement:
  […] I love photography, I take a lot of pictures, […]; it's my way of expressing myself as well. And I sit and write. (PT11)
(2) Reflecting
  Living with a chronic illness entailed a loss of abilities and capabilities that were previously taken for granted, such as abundant physical and mental energy, memorizing, pursuing a successful career or having a slim, well‐trained body. Writing can break endless ruminating cycles and release emotions, at least temporarily.
  Yes, I think [writing can give release from ruminating], on some things at least. And even with this [illness], if you feel bad or if you get annoyed with someone, or you are pissed off about something, then you write it down. And [you think] yes, to handle this is oh so hard. And then [you] kind of let go, it's released. (PT9)
(3) Tracking
  Tracking comprised the mundane activity of recording symptoms such as blood pressure, headaches or body temperature and daily activities, such as going to work or working out, or future tasks or appointments. Tracking was undertaken in apps, analogue calendars or notebooks.

#### 3.3.2 Striving for control

Information gathered through creating, reflecting and tracking actions is contextualized to gain control. Because phases of relative well‐being alternate with times of poorly controlled symptoms, personal documentation enables a more comprehensive overview of how symptoms and emotions fluctuate over extended periods, and how daily activities might influence health and well‐being. Tracking can also help a person to situate their illness in the larger context of their biography.

(1) Making sense
  Making sense pertains to understanding and coming to terms with what has happened in the past. Personal documentation allows a person to compare, understand or even become more compassionate with themselves and the current situation. Revisiting documentation can foster sense‐making and learning for the future:
  I have to take pictures every day because it's like my diary or my way of remembering, to record things, this is for real, or this has happened. This is what I looked like. So that I can understand it. Otherwise, in a year, I do not remember at all what I looked like. Or how it was […]. (PT11)
(2) Managing symptoms and emotions
  Connecting activities such as physical, social or work activities and symptoms can provide an overview of the illness trajectory and may reveal causal relationships between actions and their effect over time:
  I write, for example, if I had a very severe headache, I have had problems with that quite a lot. And then I can write down when I have a headache or if I'm tired one day, or if I'm feeling good one day and so on. So I can go back. It's also like that, and you feel that, oh my God, now I've had a headache every single day, [at least] that's what it feels like. And then you look at [your notes and realize] no; I was actually feeling good, I did this or that. Or I see a pattern, well, but now I have done quite a few things, maybe that's why I'm tired, and I should take it a little easier. (PT11)
  In times of relative well‐being, documentation may become less important:
  So you do not write on these good days, because you are busy feeling good and having a good time. And that's a good sign […] I have not written so much lately. (PT9)
(3) Managing (one's) role in society
  Personal documentation can be vital to managing one's role as a member of society. Being able to refer to written documentation about past activities becomes vital when living with severe memory loss. Personal documentation becomes ‘proof’ of productivity:
  If I have not written things down, then I have no idea what to do. […] I check the diary in the kitchen; I check my little book. I check to see what the day looks like for me. Then I have an idea of how the day will be, and I eat (breakfast) in peace. (PT6)

#### 3.3.3 Sharing

Sharing occurs at the intersection of *Gaining insight* and *Striving for control*. Whether, what, to what extent, and with whom to share personal documentation is an individual decision as it incurs the risk of making oneself vulnerable through disclosure. However, mindful sharing of personal documentation with selected persons can be beneficial.

Sharing personal documentation with healthcare professionals can support care planning or getting a diagnosis in the first place:

But before, before, [the diagnosis] it was mainly to understand what was happening and also then be able to get help when I was not really believed [by physicians] that I was actually feeling bad. Therefore, I documented, to be able to show, this is my everyday life right now, it is something that is not right, with these symptoms. (PT12)

Sharing diaries or memories with family and friends fosters feelings of connection. Digital media such as Instagram or instant messaging services even enables ‘real‐time sharing’ of messages about their experiences with friends or family:

Yes, but this was probably when I felt the worst, and then when I lived in Y. and my family lived in Z. It [writing instant chat messages] is an easy way to keep in touch. And I talked a lot with my brother when I felt so bad, and with my best friends. If you could not see each other, then you could always sort of write with them. (PT12)

In turn, reactions to posts on digital media can be rewarding:

Yes, but for now [a post about good blood results and an indication that the medication worked] there was a bit of cheering, like ‘oh how good’, ‘nice to hear’, ‘good results’ or like, positive answers. And then, as before I was to have surgery and after the operation, I also posted and then I also got comments like this, hugs, hearts and a few positive words for strength. (PT11)

## 4 DISCUSSION

This study sheds light on the role of documentation in managing life after surgery for pituitary adenoma. Based on our current findings, we propose a theory grounded in the analysis of 12 in‐depth interviews. The theory explains how three interrelated processes described as *Gaining insight, Striving for control* and *Sharing* enable *Exercising autonomy* in managing life after pituitary adenoma surgery.

The three processes did not emerge as sequential, or even of equal importance from our interviews. Rather, they emerged as individual processes that, in combination, enabled the exercising of autonomy. Because this study offers the first insights into personal documentation, we cannot make claims about how and to what extent each process may be relevant for self‐management in general. However, the current results complement previous research findings on managing life with a chronic illness.^33^ Patients have been reported to mobilize resources such as attitude, willpower and creativity to manage the challenges associated with a chronic illness.^33^ Personal documentation may be considered as one particular resource amongst several other creative strategies (working at health, participating in life, connecting with other people and developing new coping strategies) that enable patients to re‐engage in a meaningful life despite a chronic illness.^33^

The core category that emerged from this analysis was ‘exercising autonomy’. We assumed an extended concept of autonomy that encompasses individual and social‐relational autonomy. In accordance with this assumption, the analysis revealed that personal documentation was not a purely private act but a resource that can support patients' social and relational contexts or functioning. Note‐taking, for example, allows patients with memory loss to keep track of their daily activities. Moreover, our findings indicated the benefits of sharing documentation for personal relationships and communication with healthcare professionals. This finding is novel. While sharing clinical notes with patients is currently widely practised and advocated,^34^, ^35^ there is, to the best of our knowledge, no published research exploring the benefits of sharing personal documentation. Further research is needed to explore the benefits of this approach and how personal documentation may be applied to improve self‐management of long‐term illness symptoms following pituitary adenoma surgery and possibly other chronic illnesses.

Some types of personal documentation are widely encouraged in the context of more patient‐oriented chronic illness management. These documentation types often include digital applications for monitoring individual parameters such as weight or blood pressure.^12^, ^13^, ^14^, ^36^, ^37^ PGHD are focused on collecting biomedical data and patient‐defined information, such as observations of daily living, for example, feelings, thoughts or behaviours.^38^, ^39^ This combination provides a more holistic overview that enables planning, treatment and management beyond biomedical symptom and sign control. However, the patients in our study freely chose the data and documentation mode, making the documentation a genuinely personal and flexible process. Personal documentation was a tool used on an as‐needed basis (as opposed to following a prescribed format). It was adapted to optimally serve patients' changing needs during periods of better or worse health. Patients who choose to keep and maintain personal documentation produce an invaluable wealth of knowledge about their illness, which, as our findings showed, is not necessarily included in care planning. The study participants were selective about what to share with whom, and how they shared information. Considering the benefits of personal documentation, whether shared or not, our findings indicate that documentation is beneficial for managing life with the chronic symptoms following pituitary adenoma surgery.

### 4.1 Transferability

This study included 12 participants living with chronic symptoms following pituitary adenoma surgery (Table 1). Depending on the type, the long‐term symptoms that occur after pituitary adenoma surgery can vary widely.^8^ In addition to comorbidities, patients must cope with unpredictable symptoms, cognitive or psychological problems, an altered physique or personality, sexual dysfunction, fatigue, pain and difficulties regulating emotions.^10^, ^11^ The range of long‐term symptoms associated with the illness may make our findings apply to other patient groups living with a chronic illness. However, further studies are needed to confirm the theory derived from this study.

### 4.2 Limitations

This study has some limitations. First, we explored our research questions by recruiting a theoretical sample of participants who used personal documentation. Excluding participants who did not use personal documentation prevented an exploration of the potential benefits of ‘non‐documentation’. However, this type of exploration exceeded the scope of our research question. Second, we recruited our participants from a study cohort of patients living with long‐term symptoms following a pituitary adenoma. The patients' age range was 35–58 years (mean 54 years). Including younger participants (20–30 years old) may have provided more information regarding social or digital media use. Instead, we obtained an overview of the use and benefits of various media (pen and paper, digital media).

Our theoretical sampling strategy included some participants who had started documenting of their own accord, and some were inspired or encouraged by family members of healthcare professionals. Some participants had kept diaries before their diagnosis, while others started documenting after their diagnosis. Thus, our findings entail a risk of bias that is inherent in the theoretical sampling process. Our results came from participants with the motivation, skills and creativity to develop an individualized approach to their illness management. Finally, the theory was generated from a small theoretical sample and may therefore not be applicable to patients with fewer resources to self‐manage their illness.

## 5 CONCLUSION

This study sheds light on the role of personal documentation in managing the chronic symptoms following pituitary adenoma surgery. We propose a theory that explains how three interrelated processes (*Gaining insight, Striving for control* and *Sharing)* enable *Exercising autonomy* in managing daily life with a potentially serious symptom burden. Future research should elucidate whether our theory translates to other patient groups and develop tools adapted to different needs, preferences and abilities to promote personal documentation.

## AUTHOR CONTRIBUTIONS

**Birgit Heckemann**: Conceptualization; data curation; investigation; formal analysis; project administration; supervision; visualization; writing – original draft preparation; writing – review and editing (all lead). **Tatjana Graf**: Data curation; formal analysis; validation; investigation; writing – original draft preparation; writing – review and editing. **Sofie Jakobsson**: Conceptualization; formal analysis; supervision; validation; writing – original draft preparation; writing – review and editing. **Eva Jakobsson Ung**: Conceptualization; formal analysis; supervision; validation; funding acquisition; writing – original draft preparation; writing – review and editing. **Oskar Ragnarsson**: Writing – original draft preparation; writing – review and editing. **Daniel S. Olsson**: Writing – original draft preparation; writing – review and editing. **Christina Blomdahl**: Conceptualization; investigation; data curation; validation; writing – original draft preparation; writing – review and editing.

## CONFLICT OF INTEREST

The authors declare no conflict of interest.

## Data Availability

The data that support the findings of this study are available from the corresponding author upon reasonable request.
